# Tenotomy in Fractures

**Published:** 1902-05-31

**Authors:** 


### TENOTOMY IN FRACTURES.

At a recent meeting of the Liverpool Medical Institution Mr. Thelwall Thomas drew attention to the value of tenotomy of the tendo Achillis in complicated fractures of the lower extremities. In fractures of the femur above the condyles, in oblique fractures of the tibia, and in fractures of the tibia and fibula immediately above the ankle joint he had practised this operation with success. He insisted on the disablement which resulted from any deformity which distorted the line of weight bearing in the limb or altered the relative position of the joints to this axis, and held that it was essential that every fracture should be accurately reduced soon after the injury. This he thought could be readily effected by aid of tenotomy in the above-mentioned fractures, and thus the necessity for extreme flexion or for more serious operations would be diminished.

